# Acidification of drinking water improved tibia mass of broilers through the alterations of intestinal barrier and microbiota

**DOI:** 10.5713/ab.21.0455

**Published:** 2022-01-04

**Authors:** Huaiyong Zhang, Yujun Guo, Ziyang Wang, Yongshuai Wang, Bo Chen, Pengfei Du, Xiangli Zhang, Yanqun Huang, Peng Li, Joris Michiels, Wen Chen

**Affiliations:** 1 College of Animal Science and Technology, Henan Agricultural University, Zhengzhou, 450002, China; 2 Novus International, Shanghai, 200080, China; 3 Laboratory for Animal Nutrition and Animal Product Quality, Department of Animal Sciences and Aquatic Ecology, Ghent University, Ghent 9000, Belgium

**Keywords:** Acidified Water, Broiler, Intestinal barrier, Microbiota, Tibial Mass

## Abstract

**Objective:**

Diet acidification supplementation is known to influence intestinal morphology, gut microbiota, and on phosphorus (P) utilization of broilers. Alterations in intestinal barrier and microbiota have been associated with systemic inflammation and thus regulating bone turnover. Hence the effect of acidifier addition to drinking water on tibia mass and the linkages between intestinal integrity and bone were studied.

**Methods:**

One-d-old male broilers were randomly assigned to normal water (control) or continuous supply of acidified water (2% the blend of 2-hydroxy-4-methylthiobutyric acid, lactic, and phosphoric acid) group with 5 replicates of 10 chicks per replicate for 42 d.

**Results:**

Acidification of drinking water improved the ash percentage and calcium content of tibia at 42 d. Broilers receiving acidified water had increased serum P concentration compared to control birds. The acidified group showed improved intestinal barrier, evidenced by increased wall thickness, villus height, the villus height to crypt depth ratio, and upregulated *mucin-2* expression in ileum. Broilers receiving drinking water containing mixed organic acids had a higher proportion of *Firmicutes* and the ratio of *Firmicutes* and *Bacteroidetes*, as well as a lower population of *Proteobacteria*. Meanwhile, the addition of acidifier to drinking water resulted in declined ileal and serum proinflammatory factors level and increased immunoglobulin concentrations in serum. Concerning bone remodeling, acidifier addition was linked to a decrease in serum C-terminal cross-linked telopeptide of type I collagen and tartrate-resistant acid phosphatase reflecting bone resorption, whereas it did not apparently change serum alkaline phosphatase activity that is a bone formation marker.

**Conclusion:**

Acidified drinking water increased tibia mineral deposition of broilers, which was probably linked with higher P utilization and decreased bone resorption through improved intestinal integrity and gut microbiota and through decreased systemic inflammation.

## INTRODUCTION

Alterations in gait due to leg weakness and lameness causing a reduction in mobility, that may be associated with pain and a reduction in normal behaviors are important welfare and economic issues in poultry industry. In addition to decelerating weight gain to balance the growth rate and bone quality, enhancing bone mass and mechanical properties through dietary calcium (Ca) and vitamins could contribute to a lower incidence of leg problems of birds [1–3], implying that optimum Ca and phosphorus (P) in bone mineralization is directly related to leg health. The absorption of Ca and P depends on multiple factors such as the dietary concentration and forms of these minerals, passage rate of feed and viscosity of digesta, physiological environment of gut, and their interactions each other, etc. [4]. Among them, the improvement of Ca and P utilization highly rely on the pH of gastrointestinal tract which in turn is modified with dietary acidifiers [5]. It was reported that dietary supplementation to broiler chickens of 3% citric acid along with microbial phytase enzyme decreased intestinal tract pH and consequently produced better ileal nutrient digestibility and increase mineral retention such as Ca and total P [6]. Similarly, higher Ca and P blood concentrations were observed in chicks due to the dietary supplementation with organic acids such as acetic, citric, and lactic acid, and further demonstrated that the beneficial effects of organic acids were due to the lowering of gut pH and the increasing absorption of these macro-elements [7]. Consequently, when compared to control diets, dietary supplementation of citric acid was noticed to increased tibia ash percentage in broilers [8] and 64-week-old laying hens [9]. A diet with 400 mg/kg organic acid was found to increase the weight, length, and contents of Ca and P in tibia of broilers [10]. However, some workers found that organic acids had no effects on the geometrical indicators of femur and tibia in laying hens [11]. Supplementing of citric acid significantly increased percentage of bone ash of broilers, but the beneficial role was not observed in these diets with malic acid or fumaric acid [12]. Thus, a further understanding of acidifier and bone quality in broilers is required.

Recent research in our team has thrown new light on the causes of the leg problems in poultry, i.e., impairment of gut integrity and dysbiosis could induce systemic inflammation to stimulate bone resorption with the consequent result in inferior bone quality of broiler, which is termed the “gut-bone” axis [13]. It is supported by the fact that increased intestinal permeability observed in diseases, such as inflammatory bowel disease, is correlated with bone loss [14]. Previous study pointed out that chickens infected with *Salmonella* benefit from high-molecular-weight polymer (MDY), a high-molecular-weight polymer, and do not loose trabecular bone as compared to untreated birds [15]. Moreover, by comparing germ-free mice with conventionally raised mice it was shown that the presence of microbiota led to lower trabecular and cortical bone mass [16], which was accompanied by a higher number of osteoclasts and lower level of interleukin (IL)-6, tumor necrosis factor alpha (TNF-α), and CD4^+^T cells in bone [17,18], suggesting that the interaction between gut microbiota and the immune system may play a significant role in bone metabolism. More important, a study with weaned piglets found that the transcription of tight junction protein (TJPs) and *mucin-2* were upregulated by the blend of benzoic acid and *bacillus* coagulans after infecting with *Escherichia coli*, and further depressed interleukin-1β (IL-1β) and TNF-α concentration in serum and jejunal mucosa [19]. Evidence showed that administration of acidifiers induced a higher concentration of serum protein and albumin in layer chickens [20], increased the immunoglobulin A (IgA) level of the ileal and duodenal mucosa in 42-d-old broilers [21], and improved serum IgG and lymphocyte level in sucking piglets and lactating sows [22]. Besides, diet containing mixed organic acids was also associated with the change of gut microbiota, i.e., the pigs fed mixed organic acids showed a higher proportion of *Firmicutes* and a lower population of *Bacteroides*, and consequently an increase in the ratio of *Firmicutes* and B*acteroidetes* [23]. It has been shown that a decreased *Firmicutes*/*Bacteroidetes* ratio is directly related to lower inflammatory state and declined bone loss of mice [24]. Accordingly, it is reasonable to assume that the acidifier treatment is probably an important contributor for a strengthened intestinal barrier and improved gut microbiota and with a decreased inflammatory response and improved tibia mass of broilers.

Considering the side-effect of dietary acidifier on machine corrosion, moisture absorption and acid volatilization during the process of granulating or storing feed, the addition of organic acids via drinking water could avoid these problems. Therefore, this study was designed to determine the effect of drinking water supplemented with the blend of 2-hydroxy-4-methylthiobutyric acid (HMTBa), lactic, and phosphoric acid on tibia mass, intestinal barrier, microbiota, and immune status of broilers. This study is also aimed to highlight the importance of microbiota composition and intestinal integrity in regulating bone quality.

## MATERIALS AND METHODS

Animal care The experiment was conducted in a commercial farm (Zhengzhou, Henan, China) and was approved by the animal care committee of Henan Agricultural University (approval No. HNND20190612).

### Birds, housing, and diets

All birds were housed in floor pens (1.0 m×0.9 m) in a climate-controlled facility with the initial ambient temperature set at approximately 34°C. Thereafter, the temperature was gradually reduced based on normal management practices to 22°C by 20 d. A 24-h lighting regime was carried out during the first 3 d, and 23 h of lighting with 1 h of darkness was used from 4 d of age onward. Birds were vaccinated in the hatchery against Marek’s disease vaccine at 1 d, Newcastle disease at 7 d of age, and infectious bronchitis disease at 21 d of age, respectively.

One hundred 1-d-old male broiler chicks (Arbor Acres) with similar body weight (BW) were randomly distributed to normal water (Ctrl) or acidifier group with 5 replicates of 10 birds each. Acidifier was supplemented via drinking water using a liquid acidifier (Novus International Co., Ltd., consisting of 44% HMTBa, lactic and phosphoric acid as active ingredients). The pH was determined using pH meter (Hanna Instruments, Inc., Woonsocket, RI, USA), and showed that the pH of water reduced from 6.65 to 3.15 in the acidified water. The whole experimental period lasted 42 d, composed of a starter period (1 to 21 d) and a finisher period (22 to 42 d). The basal diet was formulated to meet the nutrient requirements of the National Research Council (1994) [25] and were supplied as pellets (Table 1). Each pen was equipped with a separate feeder and a nipple drinker. The water consumption was recorded at 7:00 am and 19:00 pm every day. The BW and feed intake (FI) by pen were recorded during the trial period.

### Sample collection and procedures

One bird with the average weight of each pen was sampled on d 42. Blood samples (10 mL) were taken via the jugular vein, and centrifugated at 1,500 g for 15 min at 4°C. Serum was extracted immediately and stored in −80°C until analyzed. Thymus, spleen, and bursa of Fabricius were excised and weighed, and calculated as relative weight of organ (g/100 g BW). Mid-duodenal and mid-ileal mucosa, and caecal contents were collected, then snap frozen in liquid nitrogen and stored (−80°C) suspending determination. Mid-ileum (1 cm adjacent to mid-ileum) was dissected and rapidly immersed in phosphate-buffered formaldehyde for histology analysis. The right tibiae were removed for length, bone weight, and mineralization properties measurement.

### Fat-free weight and mineral content of tibia

The tibia of each bird was air-dried for 24 h at room temperature, extracted by ethyl ether for 48 h, oven-dried at 108°C for 24 h for dry fat-free bone weight determination. Subsequently, dry-defatted tibia was ashed in a muffle furnace at 550°C for 24 h and the ash was measured based on the percentage of dry-defatted weight. Ca and P contents were determined through ethylene diamine tetraacetic acid titration and ammonium metavanadate colorimetry, respectively, and values were also presented based on dry fat-free weight [26].

### Ileal morphology

Formalin-fixed ileal samples were dehydrated, embedded, sliced into 5-μm transects, and stained with hematoxylin and eosin, and subsequently wall thickness, villus height and crypt depth of at least ten well-oriented villi, were measured and the ratio of villus height to crypt depth was calculated [27].

### Gut microbiome

Total bacterial genomic DNA was extracted from digesta samples of cecum by use of the Stool DNA Kits (Invitek, Westburg, the Netherlands). After evaluation of DNA concentration and purity, the V3–V4 hypervariable region of the bacterial 16S rRNA was amplified using the specific primer (F: 5′-CCTACGGGRSGCAGCAG-3′; R: 5′-GGACTACV VGGGTATCTAATC-3′). The 16S rDNA high-throughput sequencing was performed using the Illumina platform (Illumina, San Diego, CA, USA). The obtained sequences were processed for alignment and cluster into operational taxonomic units (OTUs) at 97% similarity using USEARCH (v7.0.1090) in QIIME software. The alpha diversity was evaluated by calculating the Chao1, Shannon, and Simpson index using QIIME software. Beta-diversity at genus level was estimated by calculating Bray-Curtis dissimilarity and visualized with principal co-ordinates analysis [27].

### Cecal short-chain fatty acid analysis

Cecal contents were weighted, and approximately 1 g was dissolved in 5.5 mL 10% formic acid containing 0.5 mg ethyl butyric acid as the internal standard. After filtration and centrifuging the supernatants were used to determine total short-chain fatty acid (SCFA), acetate, propionate, butyrate, iso-butyrate, valerate, and iso-valerate concentrations using a gas chromatography on a Shimadzu 2010 (Shimadzu Corporation, ‘s-Hertogenbosch, The Netherlands) equipped with a flame ionization detector. The conditions were used as described previously [28].

### Blood biochemistry

Serum Ca, P concentration was determined with Biochemistry Analyzer (Yellow Springs Instrument Co. Inc., Yellow Springs, OH, USA) using *o*-cresol phthalein and ammonium molybdate, respectively. Serum bone turnover markers, C-terminal cross-linked telopeptide of type I collagen (CTx) was measured using an immunoassay (Cobas; Roche Diagnostics, Basel, Switzerland). Alkaline phosphatase (ALP) was measured by catalyzing the hydrolysis of colorless *p*-nitrophenyl phosphate to give *p*-nitrophenol using commercially available assay kits. Tartrate-resistant acid phosphatase (TRAP) activity concentration was assayed by the enzyme-linked immunosorbent assay (ELISA). The kits for ALP and TRAP were obtained from Nanjing Jiancheng Bioengineering Institute (Nanjing, China). Concentrations of serum IgG, IgM, and IgA were assayed using Chicken ELISA Quantitation Kits (Bethyl Laboratories Inc., Montgomery, TX, USA). The levels of TNF-α, IL-1β, and IL-6 in serum were measured with commercially available kits (Mosak Biotechnology Co., Ltd, Wuhan, China). These assays were performed as per procedures described by the manufacturers. Each sample was run in duplicate

### Quantitation of mRNA using real-time polymerase chain reaction

Relative quantification of mRNA levels of intestinal barrier, inflammatory cytokine, and the transporter of Ca and P were performed by real-time polymerase chain reaction (RT-PCR). After extracted total RNA and inverse transcription, the obtained cDNA was amplified by 40 cycles (1 cycle: 95°C for 30 s, annealing at 60°C for 34 s) and a final melting curve analysis in ABI 7500 Real-Time PCR detection system (Applied Biosystems, Warrington, UK). Primers were designed using online Primer 3 and are shown in Table 2. Values were normalized to glyceraldehyde-3-phosphate dehydrogenase (*GAPDH*) and *β-actin*.

### Statistical analysis

The results were expressed as mean±standard deviation. Statistical power of 0.80 (80%) was obtained in this study when the minimally detectable effect size was 1.0 and the significance level was 0.05. Data were checked for normal distribution and homogeneity of variance using the Shapiro-Wilk and Levene’s tests, respectively, in SAS statistical software (version 9.2, SAS Institute, Cary, NC, USA). Two-tailed unpaired t-test or the Mann-Whitney U test for normally or non-normally distributed data were used to evaluate the statistical differences of biological parameters between control and acidifier groups, respectively.

## RESULTS

### Growth performance and water consumption

**Figure 1:**
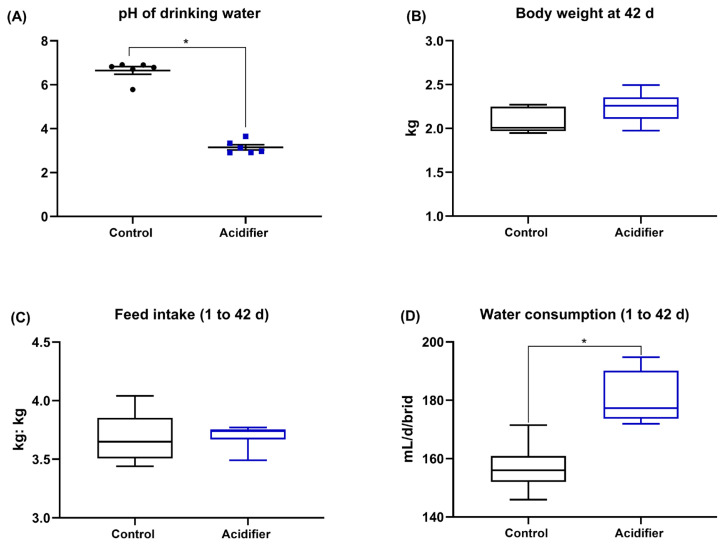
The pH of drinking water used in the present study (A), and effect of acidification of drinking water in broilers on (B) body weight, (C) feed intake, and (D) water consumption. * Denotes significant difference between Ctrl and acidifier group at p<0.05.

As presented in Figure 1A, the supplementation with acidifier to drinking water notably decreased the pH of water (p<0.01). The experimental treatments had no significant effect on BW and FI (p>0.05). However, the broiler chickens fed acidified water had a higher water consumption compared with the control birds (p<0.05; Figure 1B–1D).

### Tibial quality

**Figure 2:**
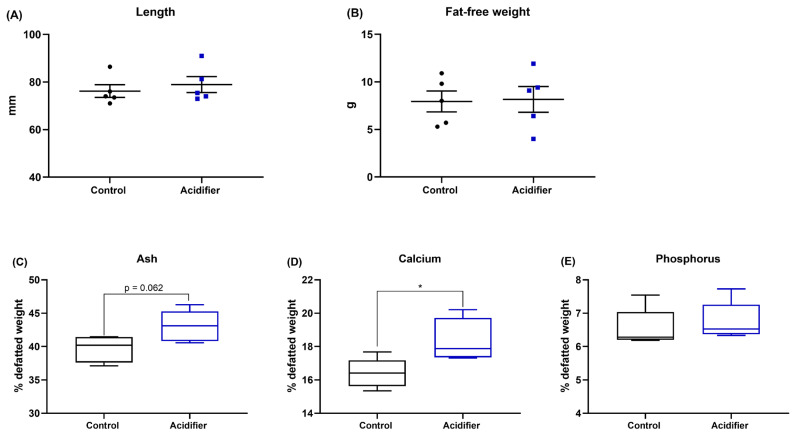
Effect of acidification of drinking water on (A) bone length, (B) fat-free weight, (C) tibia ash, (D) calcium content, and (E) phosphorus content of 42-d-old broilers. * Denotes significant difference between Ctrl and acidifier group at p<0.05.

The results of supplementation of acidifier via drinking water on tibial characteristics of broiler chickens are summarized in Figure 2; bone growth was not affected by the experimental treatments, indicated by comparable bong length and fat-free weight of tibia (p>0.05; Figure 1A–B). Data of bone mineralization manifested by those birds in the acidifier group had a numerical increase in tibia ash (p = 0.062) and a significant increase in Ca content (p<0.05). Whereas there was no significant difference between treatments on the concentration of P in broiler chickens at 42 d of age (Figure 1E).

### Intestinal barrier

**Figure 3:**
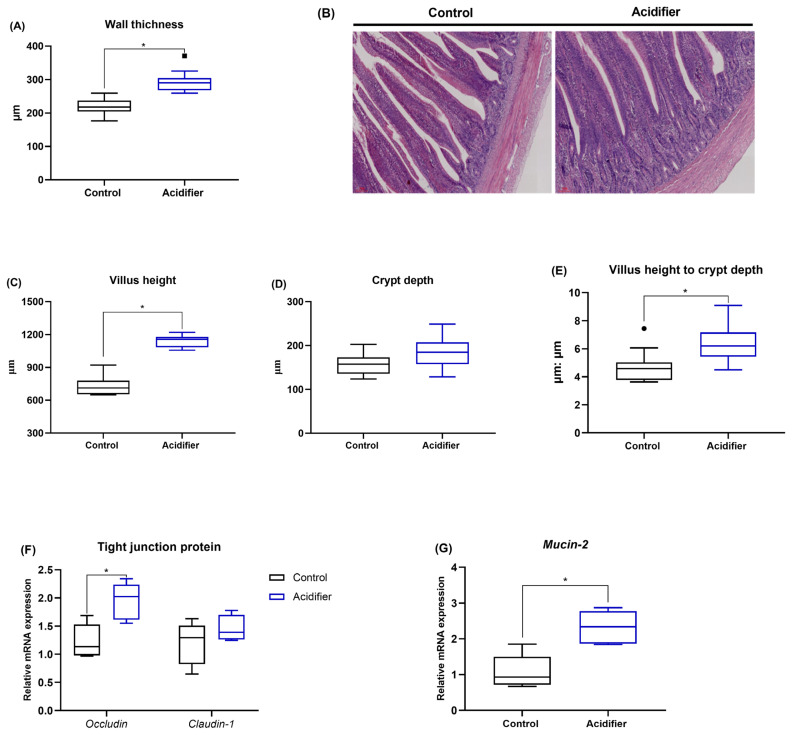
Effect of acidification of drinking water on intestinal barrier of ileum in 42-d-old broilers. (A) Wall thickness, (B) hematoxylin and eosin (H&E) staining (×100), (C) villus height, (D) crypt depth, and (E) their ratio, mRNA abundance of (F) *mucin-2* and (G) *occludin* and *claudin-1*. * Denotes significant difference between Ctrl and acidifier group at p<0.05.

The results of the effects of experimental treatments on intestinal morphology in broiler chickens are shown in Figure 3A–3E. Supplementing with acidifier significantly increased the wall thickness, villus height, and the ratio of villus height and crypt depth (p<0.05), but it did not affect crypt depth in ileum. Analysis of the intestinal barrier genes shows that the mRNA abundance of *occluding*, *claudin-1*, and *mucin-2* were increased by acidified water as compared with control group (Figure 3F, 3G).

### Caecal microbiome

**Figure 4:**
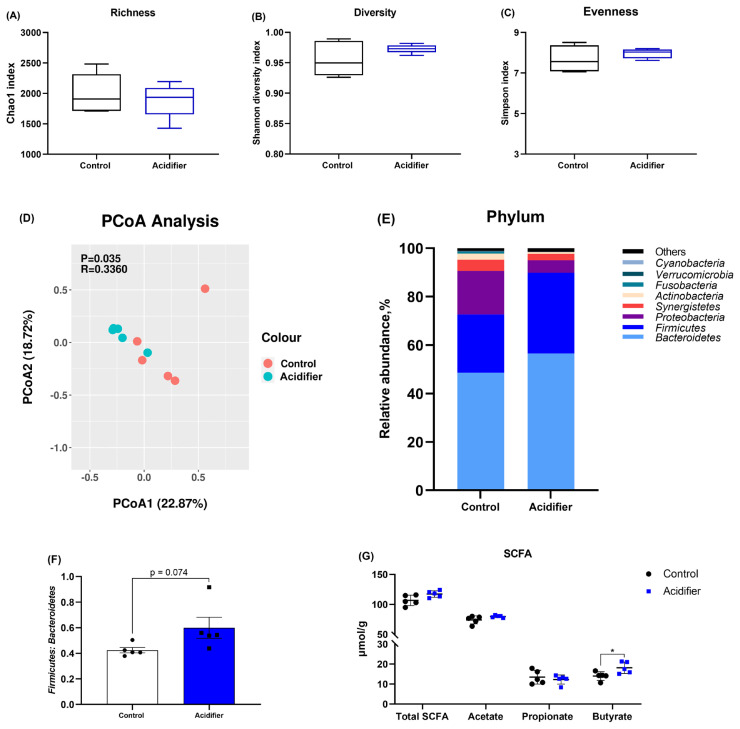
Effect of acidification of drinking water in broilers at d 42 on caecal microbiome; (A–C) Chao1 and Simpson indexes were used to assess diversity and evenness, (D) principal coordinate analysis plot (PCoA) of caecum microbiome diversity at species level based on Bray-Curtis dissimilarities, (E) relative abundances of bacterial communities at phylum level, (F) the *Firmicutes* to *Bacteroidetes* ratio (G) caecal short-chain fatty acid (SCFA). * Denotes significant difference between Ctrl and acidifier group at p<0.05.

Concerning the effects of acidifier on the caecal microbiome, the alpha diversity was not notably changed by experiment treatment, indicated by comparable Chao 1, Shannon, and Simpson indexes (p>0.05; Figure 4A–4C). The samples in the acidifier group formed a distinct cluster from those in the control group (Figure 4D). The compositions of caecal microbiota at phylum level differed between the two groups, and the caecal microbiota in broilers was dominated by the *Firmicutes* and *Bacteroidetes* phyla (Figure 4E). *In concreto*, drinking water containing mixed organic acids shows a higher proportion of *Firmicutes*, *Bacteroidete*, and the ratio of *Firmicutes* and *Bacteroidetes*, as well as a lower population of *Proteobacteria* compared with control group (Figure 4E, 4F).

Total and individual SCFA contents of the caeca are also given in Figure 4G. Experimental manipulation did not result in changes in terms of the levels of total SCFA, acetate, and propionate (p>0.05). However, acidified water notably increased butyrate concentration when compared with control group (p<0.05).

### Immune status

**Figure 5:**
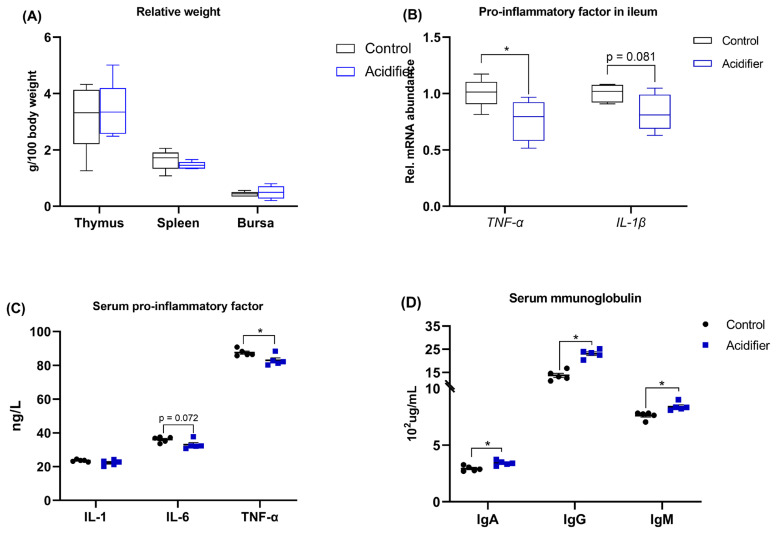
Effect of acidification of drinking water in broilers at d 41 on immunological indices; (A) relative weight of thymus, spleen, and bursa, (B) the mRNA abundance of pro-inflammatory cytokines including interleukin (IL)-1β and tumor necrosis factor-α (TNF-α) in ileum. (C) Serum concentration of IL-1, IL-6, and TNF-α, as well as (D) the level of immunoglobulin (Ig) including IgG, IgM, and IgA. * Denotes significant difference between Ctrl and acidifier group at p<0.05.

As illustrated in Figure 5. There was no significant difference among treatments on the relative weight of thymus, spleen, and bursa in broilers at 42 d of age (Figure 5A). The data of inflammation analysis revealed that the mRNA levels of proinflammatory factors, including *TNF-α* and *IL-1β*, were decreased in the ileum of acidifier-treated birds compared with control broilers (Figure 5B). Reflecting to serum, birds receiving acidified water exhibited a decreased level of IL-6 (p = 0.072) and TNF-α (p<0.05), but similar IL-1 level when compared to control birds (Figure 5C). Furthermore, Serum IgG, IgA, and IgM concentrations were increased by acidifier administration (p<0.05; Figure 5D).

### Ca and P absorption

**Figure 6:**
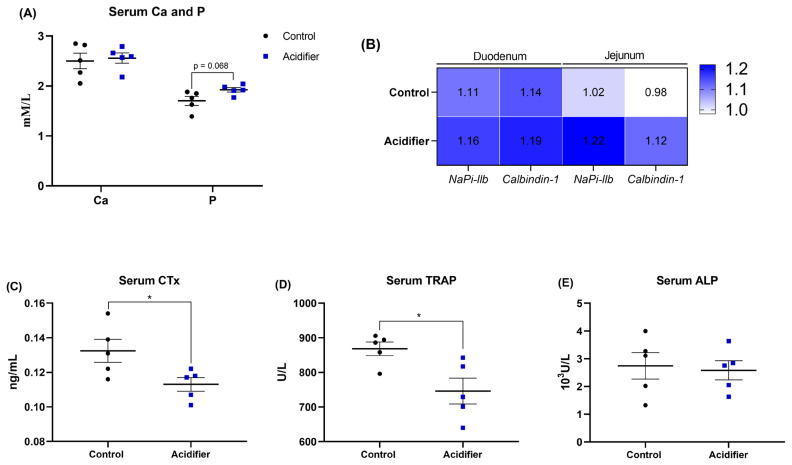
Effect of acidification of drinking water in 41-d-old broilers on Ca and P homeostasis and bone turnover. (A) Serum calcium and phosphorus content. (B) mRNA abundance of real-time polymerase chain reaction analysis for mRNA expression of sodium-dependent phosphorus transport protein II (*NaPi-IIb*) and *calbindin-1* in duodenum and ileum. In addition, serum bone resorption biomarker (C) C-terminal cross-linked telopeptide of type I collagen (CTx) and (D) tartrate-resistant acid phosphatase (TRAP) level, as well as bone formation biomarker alkaline phosphatase activity (ALP) were determined. * Denotes significant difference between Ctrl and acidifier group at p<0.05.

Effects of acidified water on Ca and P absorption are shown in Figure 6A, 6B. Serum P concentrations tend to increase (p = 0.068) due to acidifier treatment, whereas serum Ca concentrations were unaffected (p>0.05). No difference was also observed between acidifier and Ctrl groups in terms of the mRNA expressions of *calbindin-1* and sodium-dependent phosphorus transport protein IIb (*NaPi-IIb*) in the duodenum but not jejunum, in which acidified water result in a numerical increase in the transcription of *calbindin-1* and *NaPi-IIb* when compared with Ctrl group (Figure 6B).

### Bone turnover

Experimental administration significantly affected the bone resorption, showed by the decreased circulatory level of CTx and TRAP, both reflect bone resorption, in acidifier group compared with control birds (p<0.05; Figure 6C, 6D). Regarding bone formation, no obvious differences in the serum ALP activity, an indicator of bone formation, between acidifier and control groups (p>0.05; Figure 6E).

## DISCUSSION

Acidification of drinking water has been previously confirmed to improve the performance of broilers [29] and laying hens [30]. The current study also showed that water acidification induced higher water consumption and thus improved BW of broilers, which may be attributed to that the organic acids and their salts promotes protein and energy digestibility [31] and/or stimulates pancreatic enzyme secretion and activity [32]. In addition, the diet with 400 mg/kg organic acid was found to increase the weight, length, and contents of Ca and P in tibia of broilers [10]. Supplementation of citric acid was also noticed to increased tibia ash percentage as compared with unsupplemented diets in broilers [8] and 64-week-old laying hens [9]. Findings corresponding to our results were obtained by Mohammadpour et al [33] who indicated that bone density and ash percentage of tibia but not weight, volume, and length of tibia in 21-d-old broilers were increased after the addition of citric acid. In line with these results, Świątkiewicz et al [11] found that organic acids had no significant effects on the geometrical indicators of femur and tibia in laying hens. This discrepancy could be partly explained by the form and dose of acidifier and the suboptimal conditions of nutrients such as dietary P level [34]. Some workers indicated that the beneficial effect of acidifier on growth and tibia ash depended on dietary P level, i.e. the tibia ash of broilers was maximized at lower available P levels when the diets comprised 4% or 6% citric acid as compared to the diets containing no citric acid [2]. Moreover, the dietary addition of citric acid but not malic acid or fumaric acid significantly increased percentage of bone ash of broilers [12]. Collectively, the results of the present study indicated that acidifying drinking water had some beneficial effects on BW and tibia mineralization in 42-d-old broilers.

To understand the mechanisms linking changes by acidifier to bone quality, it is useful to consider the two potential mechanisms: regulation of nutrient absorption, especially Ca and P [23,31], regulation of the intestinal barrier and gut microbiome [23,35,36], and consequently interfering this link between the gut and bone remolding mediated by immune system [37,38]. It was reported that dietary citric acid could effectively improve the utilization of phytate P in laying hens [39]. Dietary supplementation of 3% citric acid along with microbial phytase enzyme in broiler chicken was noticed to produce better ileal nutrient digestibility and increase mineral retention such as Ca and total P, and authors pointed out that improved P absorption was associated with lower pH of gastrointestinal tract facilitating the P solubility [6]. The organic acid supplementation together with the developing desirable gut microflora was deemed to contribute mineral retention and bone mineralization through increased digestibility and availability of nutrients [10]. Accordingly, a remarkably higher Ca and P blood concentrations were observed in chicks fed a diet supplemented with organic acids such as acetic, citric, and lactic acid, and further demonstrated that the beneficial effects of organic acids were caused by the lowering of gut pH and the increase in the absorption of these macro-elements [7]. By contrast, it has been mentioned that formic acid at the levels of 0.5% or 1% did not affect serum Ca and P levels in chickens raised under good hygiene conditions [40]. Manipulation with a 3% inclusion of citric acid in diet did not change blood Ca and P concentrations of in broiler chickens [41]. These variations might be regarded to feed ingredients and management conditions, as well as the endocrine regulation for maintaining the homeostasis of Ca and P [42]. In the present study, Ca content in serum was not influenced by experimental treatments, whereas serum P concentrations tend to increase due to acidified water, which was probably due to the upregulated transcription of *NaPi-IIb*, a regulator of P transport in intestine [43]. In addition, studies have also reported that acidifier induced morphological changes to the small intestine of broilers mainly consist of an increased villus height and villus/crypt ratio of ileum [35]. Inevitably, this will lead to substantial augment of intestinal digestion and absorption surface. This is consistent with our results on the ileum histomorphology of birds in this study. Furthermore, the increases in villus height by supplementation with acidifier in drinking water reflects enhanced digestion and absorption capacities in broilers.

Impairment of gut integrity and dysbiosis, and consequent regulation of systemic immune function might be another likely reason for the differences in bone quality in the current study. The TJPs serve as the innate defense barrier, formed by zonula occludens-1 (ZO-1), claudins, occludin, and adherence junctions, along with the mucus that covers its surface. These protect the host against paracellular bacterial infiltration and penetration of toxic substrates [44]. Our results indicated acidification of drinking water enhanced intestinal barrier of ileum, instructed by upregulated expression of *occludin*, *claudin-1*, and *mucin-2* mRNA abundance, which were in line with findings in weaned piglets saying that the mRNA abundance of *claudin-1*, *occludin*, *ZO-1*, and *mucin-2* was upregulated by the blend of benzoic acid and *bacillus* coagulans after infecting with *Escherichia coli* [19]. These alterations of intestinal permeability can be related to bone health, supported by the fact that increased intestinal permeability observed in diseases, such as inflammatory bowel disease, is correlated with bone loss [14]. The high-molecular-weight polymer MDY is known to protect intestinal epithelial integrity against injury. A previous study pointed out that chickens infected with *Salmonella* benefit from MDY and do not loose trabecular bone as compared to untreated birds [15]. More important, in this study, acidified water caused significant changes in microbial composition as evidenced by forming a distinct cluster that was separated from control group. Relative abundance at the phylum levels manifested in substantial increases in *Firmicutes* and *Bacteroidetes*, and a decrease in *Proteobacteria*. A decrease in *Bacteroidetes* abundance has been associated with intestinal inflammation such as inflammatory bowel disease (IBD) [45]. Reduced *Firmicutes* to *Bacteroidetes* ratio was linked with lower inflammatory state and declined bone loss of mice [24]. In addition, data has indicated that the proportion of *proteobacteria* was lower in normal control than that in osteoporosis and osteopenia patients [46]. Concerning the linkage between organic acid and gut microbiota, it was reported that pigs fed a diet containing mixed organic acids showed a higher proportion of *Firmicutes* and the ratio of *Firmicutes* and *Bacteroidetes*, as well as a lower population of *Proteobacteria* [23]. Therefore, the alternation in microbiota due to acidifier manipulation in this study probably exerted a positive role in tibia mass in broilers. Of note, several reports highlighted the immunomodulatory capacities of SCFA and provided a direct mechanistic link between the gut microbiota and bone [47]. A recent review says that the improvement in bone properties were associated with increments in SCFA levels [38], which was further confirmed by the numerical increase in total SCFA concentrations and significant augment in butyrate production in the current study. In addition, the elevated butyrate could also promote intact mucosal development and barrier functions through providing energy for intestinal epithelial cell growth and inducing mucin-2 synthesis [48,49], and further enhanced the favor role of intestinal barrier on bone quality. Taken together, these results underscore the importance of acidification of drinking water on the “gut-bone” signaling axis.

Under conditions of impaired gut integrity and/or microbiota dysbiosis, bacteria and their factors can translocate across intestinal barrier to induce systemic inflammatory responses [14]. This study, using relative weigh of immune organs, the pro-inflammatory factors and immunoglobulins level in serum, and the ileum pro-inflammatory factors transcription to access the immunological status, manifested that acidifier decreased intestinal inflammation and improved the immune condition, which was supported by previous studies in broilers [7,8]. Currently, our research showed there were no significant differences on the relative weight of thymus, spleen, and bursa between acidifier and control group. Several studies in broilers elucidated those organic acids increased the weight of lymphoid organs [7] and the spleen weight [21]. A possible reason for this variable effect may be the difference in the dose and manner of acidifier used in these studies. Furthermore, acidification of drinking water improved the immune status of broilers by increasing the concentration of serum antibodies (IgA, IgG, and IgM). A previous study also revealed that administration of acidifiers markedly increased serum protein and albumin concentration in layer chickens [20], elevated the IgA level of the ileal and duodenal mucosa in 42-d-old broilers [21], and improved serum IgG and lymphocyte level in sucking piglets and lactating sows [22]. In addition, data from the ileal and serum inflammatory factors in this study suggested that acidified water decreased inflammatory response of birds consistent with findings in weaned piglets [19]. It is well-established that inflammatory cytokines seem to have a direct role on bone remodeling via influencing the recruitment, maturation, proliferation, and activation of osteoclasts [50]. In this regard, rheumatoid arthritis characterized by inflammation and bone destruction mediated by osteoclasts proved to be a case for illustrating the relationship between inflammatory cytokines and bone metabolism [51]. TNF-α, which is secreted along with IL-1 from mononuclear cells, promotes osteoclastogenesis [52]. Mice with TNF-α induced arthritis were found to have increased circulation of osteoclast precursors, and further was reversed by anti-TNF-α therapy and correlated with systemically increased TNF-α concentrations [53]. In addition, IL-6 is also another crucial inflammatory cytokine to stimulate osteoclast formation and function *in vitro*. IL-6 deficient mice were protected from a significant loss of bone mass together with an increase in bone turnover rates caused by estrogen depletion [54]. Here, the depressed expression of inflammatory factors, especially TNF-α, in ileal mucosa of broilers given acidifier implied that acidified water improvement of tibia mass probably results from depressed inflammatory-mediated bone resorption. During bone resorption, TRAP is secreted into the circulation by osteoclasts and closely associated with bone resorption [55]. CTx is peptide fragment generated by collagen degradation, and it is regarded as important biochemical markers of bone resorption [56]. In this sense, these data inferred that acidifier probably reduced bone resorption by inhibiting the release of inflammatory factors that can promote osteoclastogenesis because of impaired intestinal integrity and/or dysbiosis. Additional analyses of bone formation indicate that it was did not regulated by acidifier. To be specific, supplementation of acidifier to drinking water not apparently changed serum ALP level, because ALP is a byproduct of osteoblast activity and has been proposed as a marker of bone formation [57]. Thus, these results imply that the positive effect of acidifier on tibia mass of birds is likely mediated by suppressing bone resorption but not by stimulating bone formation.

## CONCLUSION

This study demonstrated that the blend of HMTBa, lactic and phosphoric acid as an additive in drinking water increased tibia mineral deposition of broilers and produced healthy broilers that possessed a higher immune status. Moreover, we also highlighted the importance of microbiota composition and intestinal barrier function in regulating bone quality, i.e., improving intestinal integrity and gut microbiota by acidification of drinking water led to decreased inflammation and bone resorption, and consequently enhanced tibial quality.

## Figures and Tables

**Table 1:** Composition and nutrient analysis of experimental diet (as-fed basis)

Items	1 to 21 d	22 to 42 d
Ingredients (%)		
Corn	55.00	60.48
Soybean meal	32.7	25.8
Corn protein flour	5.0	5.0
Dicalcium phosphate	1.5	1.0
Stone powder	1.1	1.0
Sodium chloride	0.3	0.3
Soybean oil	3.0	5.3
Premix^1)^	0.6	0.6
DL-methionine, 98%	0.21	0.16
L-lysine hydrochloride, 98.5%	0.46	0.31
L-threonine, 98.5%	0.13	0.05
Total	100.0	100.0
Nutrient analysis^2)^ (%)		
Metabolizable energy (kcal/kg)	3,000	3,202
Crude protein	23.20	19.78
Calcium	0.82	0.67
Total phosphorus	0.56	0.50
Lysine	1.44	1.15
Methionine	0.56	0.47
Threonine	0.97	0.78

1) Premix is provided per kilogram of diet: 1–21 d: vitamin A, 12,000 IU; vitamin D_3_, 3,500 IU; vitamin E, 60 IU; vitamin K_3_, 4 mg; vitamin B_1_, 2.5 mg; vitamin B_6_, 6 mg; vitamin B_12_, 8 μg; D-Pantothenic acid, 40 mg; Niacin, 75 mg; folic acid, 10 mg; biotin, 0.8 mg; choline, 700 mg; Cu (CuSO_4_·5H_2_O), 20 mg; Fe (FeSO_4_·7H_2_O), 100 mg; Zn (ZnSO_4_·7H_2_O), 90 mg; Mn (MnSO_4_·H_2_O), 100 mg; Se (NaSeO_3_), 0.3 mg; I (KI), 0.5 mg; phytase, 0.1 g. 22–42 d: vitamin A, 10,000 IU; vitamin D_3_, 3,000 IU; vitamin E, 50 IU; vitamin K_3_, 3.5 mg; vitamin B_1_, 2 mg; vitamin B_6_, 5 mg; vitamin B_12_, 6 μg; D-pantothenic acid, 20 mg; niacin, 60 mg; folic acid, 8 mg; biotin, 0.6 mg; choline, 600 mg; Cu (CuSO_4_·5H_2_O), 15 mg; Fe (FeSO_4_·7H_2_O), 100 mg; Zn (ZnSO_4_·7H_2_O), 80 mg; Mn (MnSO_4_·H_2_O), 80 mg; Se (NaSeO_3_), 0.3 mg; I (KI), 0.5 mg; phytase, 0.1 g

2) Metabolizable energy, methionine, lysine, and threonine in the nutritional level were calculated values, and the rest were measured values.

**Table 2:** The primers for quantitative real-time polymerase chain reaction

Gene	Gene ID	Primer	Sequence (5′-3′)	Size (bp)
*NaPi-IIb*	NM_204474.2	Reverse	tcatccatcatcgtcagcat	81
		Forward	aatgtttgcccccataatga	
*Calbindin-1*	NM_205513.1	Reverse	aggcaggcttggacttaac	97
		Forward	acctgagcaagctcaacgat	
*Claudin-1*	NM_001013611.2	Reverse	gtctttggtggcgtgatctt	117
		Forward	tctggtgttaacgggtgtga	
*Occludin*	NM_205128.1	Reverse	ccagaagacgcgcagtaaga	107
		Forward	cgttcttcacccactcctcc	
*Mucin-2*	NM_001318434.1	Reverse	tgccagcctttttatgctct	80
		Forward	agtggccatggtttcttgtc	
*IL-1β*	NM_204524.1	Reverse	gtttttgagcccgtcacct	117
		Forward	cacgaagcacttctggttga	
*TNF-α*	NM_204267.1	Reverse	agatgggaagggaatgaacc	120
		Forward	actgggcggtcatagaacag	
*β-actin*	NM_205518.1	Reverse	gctacagcttcaccaccaca	90
		Forward	tctcctgctcgaaatccagt	
*GAPDH*	NM_204305.1	Reverse	tgggaagcttactggaatgg	88
		Forward	cttggctggtttctccagac	

*NaPi-II*, sodium-dependent phosphorus transport protein II; *IL*, interleukin; *TNF-α*, tumor necrosis factor-alpha; *GAPDH*, glyceraldehyde-3-phosphate dehydrogenase.
